# Imaginary Companions in Childhood: Relations to Imagination Skills and Autobiographical Memory in Adults

**DOI:** 10.1080/10400419.2015.1087240

**Published:** 2015-11-13

**Authors:** Lucy Firth, Ben Alderson-Day, Natalie Woods, Charles Fernyhough

**Affiliations:** Department of Psychology, Durham University, Durham, UK

## Abstract

The presence of a childhood imaginary companion (IC) has been proposed to reflect heightened imaginative abilities. This study hypothesized that adults who reported having a childhood IC would score higher on a task requiring the imaginative construction of visual scenes. Additionally, it was proposed that individuals who produced more vivid and detailed scenes would also report richer autobiographical memories, due to a shared reliance on imaginative abilities in construction and recollection. Sixty participants (20 with an IC), completed an adapted scene construction procedure and an autobiographical memory questionnaire. Participants reporting a childhood IC scored significantly higher on scene construction and rated themselves as more imaginative. Scene construction scores were also moderately related to the richness of autobiographical memories, although this was almost entirely due to scores on the thought/emotion/action component of scene construction. Autobiographical memory was unrelated to the presence of an IC. Implications for overlapping and dissociable aspects of imagination and memory are discussed.

Imaginary companions (ICs) in childhood are a common occurrence (McLewin & Muller, 2006). ICs are typically reported in 10%–30% of participants in studies with children (Bouldin & Pratt, 2001; Davis, Meins, & Fernyhough, 2011; Taylor & Carlson, 1997). The presence of ICs has variously been proposed to be an early marker for psychopathology (McLewin & Muller, 2006), a characteristic of typical development (Taylor, Carlson, Maring, Gerow, & Charley, 2004), and an indicator of creativity (Gleason, Jarudi, & Cheek, 2003; Schaefer, 1969).

A number of studies have examined enhanced creative and imaginative abilities in children with an IC. Trionfi and Reese (2009) found that such children told richer, more detailed stories and showed more advanced narrative skills. Singer (1961) reported that, in the context of a game, children with ICs were more willing to wait for an extended period of time—during which they easily entered in to imaginative play—and also produced higher scores when marked on imaginativeness of storytelling. Bouldin and Pratt (2001) found that children with an IC were rated by parents as being significantly more imaginative than those who did not have an IC, along with being more inclined to fantasy and more likely to explain events as magical. Studies involving adolescents have shown similar results: Schaefer (1969) reported that, in a sample of American high-school adolescents, students who had creative achievements in the literary field were more likely to recall childhood ICs than a control group. Seiffge-Krenke (1997) found that, among adolescents, those who reported ICs engaged significantly more in imaginative activities such as day-dreaming.

In contrast to the research on children and adolescents, relatively few studies have sought to investigate whether having an IC is predictive of future imaginative abilities. Gleason et al. (2003) studied 102 female students, of whom 29.4% reported having an IC. Participants with an IC used significantly more imagery, reported more vivid night-dreams, and scored higher on a composite imagination measure (Gleason et al., 2003). Kidd, Rogers, and Rogers (2010) conducted two studies focusing on whether having an IC as a child was associated with particular personality characteristics in adulthood. They first reported that students who recalled having an IC scored higher on a self-report measure of creativity. Then, using another population of students, they showed that those with a childhood IC scored higher on the *absorption in active imagination* personality dimension (Kidd et al., 2010). Finally, Dierker, Dais, and Sanders (1995) showed that adults who reported the presence of an IC in childhood also scored highly on an imagination inventory and a measure of dissociative traits (such as absorption).

Although these studies point to an advantage in imaginative abilities for adults who had a childhood IC, they share the limitation of only using self-report measures of imagination, leaving their true explanatory value open to question. This study aimed to build on these previous investigations by assessing imagination using a new and more objective methodology: scene construction. Scene construction requires participants to imagine and describe a number of fictitious scenes, with the descriptions then being coded for levels of complexity and detail (Hassabis & Maguire, 2007). Our first hypothesis was that participants who reported having an IC as a child would produce more vivid and detailed scene constructions than those who did not.

A secondary aim was to investigate the interrelation of ICs, imagination, and autobiographical memory. Hassabis and Maguire (2007) argued that the psychological processes involved in memory and imagining fictitious experiences share striking similarities. Similar brain areas are thought to underpin episodic memory, future thinking, and imagination (Miller, 2007): Patients with damage to the hippocampus, for example, not only show memory impairments, but also impairments on a future thinking and imagination task (Hassabis, Kumaran, Vann, & Maguire, 2007). A second hypothesis was that participants who produced more vivid and detailed scene constructions would also report richer autobiographical memories. Although previous research connecting scene construction and memory focused on populations with amnesia (Hassabis et al., 2007) or schizophrenia (Raffard, D’Argembeau, Bayard, Boulenger, & Van der Linden, 2010), this study aimed to extend such findings to a nonclinical, young adult population.

## Method

### Participants

The study group consisted of 60 participants (14 men). All participants were students aged 18–23 years (*M* = 19.9 years), and were recruited through participant pool advertisements. A third (*n* = 20) of the sample reported having had a childhood IC. No participant stated that they currently had an IC.

### Materials

All participants were given a questionnaire on their imagination and memory skills. Participants had to rate their memory and imagination ability on a 10-point scale (1 = *poor* to 10 = *very good*). The questionnaire then asked if the participant previously or currently had an IC; if so, they were asked to provide a brief description.

The second part of the questionnaire focused on autobiographical memory, for which participants were asked to recall two personal happy memories: one from their childhood and one from the past year. Happy memories were chosen to avoid participants choosing something upsetting that they may have not wished to think about in substantial detail. For each memory, the participants completed a revised version of the Memory Characteristics Questionnaire (Johnson, Foley, Suengas, & Raye, 1988), comprising 23 items about the detail and vividness of the memory selected. For each item the participants had to give a score of between 1 and 7; items included details about sensory information, order of events, feelings, storyline, time, and accuracy. The maximum score possible for each memory was 161. The scores for memory 1 (childhood event) and memory 2 (event within the past year) were then summed to produce the autobiographical memory total (AMT) score.

The scene construction procedure was adapted from Hassabis et al. (2007). Participants were given four cue cards detailing the scenes they were to describe (beach, market, museum, and forest) and a brief description of what to include (e.g., “Imagine yourself standing by a small stream somewhere deep in a forest. I want you to describe the experience and the surrounding in as much detail as possible using all your senses including what you can see, hear, and feel.”).

The participant’s four descriptions were transcribed and scored separately in accordance with Hassabis et al.’s (2007) method. Each scene was segmented into a set of statements that were then classified as belonging to one of four categories: spatial reference (SR), entity presence (EP), sensory description (SD), or thought/emotion/action (TEA). The SR category referred to statements regarding the relative position of entities within the environment, directions relative to the participant’s vantage point, or explicit measurements or positions (e.g., “next to the trees” or “to my left I can see”). The EP category referred to any distinct entities (e.g., objects, places, people, animals) mentioned (e.g., “There are some stalls”). The SD category consisted of any statements describing (in any modality) the properties of an entity (e.g., “the sea is very blue”), as well as general weather and atmosphere descriptions (e.g., “it is very hot” or “it smells musty”). Finally, the TEA category encompassed any introspective thoughts (e.g., “I wouldn’t understand”) or emotional feelings (e.g., “I feel very happy”), as well as the thoughts, intentions, and actions of other entities in the scene (e.g., “my sister is swimming”). There were no maximum or minimum scores set; in contrast to Hassabis et al.’s (2007) experiment, scores were not capped at 7. For every participant, a score for each category was produced per scene and then total SR, EP, SD, and TEA scores were calculated. Finally, a total scene construction score was calculated comprised of a count of the total number of statements.

### Procedure

Participants were all tested in a private room with only the experimenter present. Participants completed the questionnaire pack and then the scene construction procedure, following Hassabis et al. (2007). Brief descriptions of the four scenes were read aloud one at a time. Participants were asked to visualize the scene and describe it in as much detail as they could, using all of their senses. They were also told that if they were struggling, they would be prompted. Cue cards detailing the scene description and instructions were also provided. To avoid order effects, scene presentation was counter-balanced. All procedures were approved by a university ethics committee.

## Results

### Imaginary Companions and Scene Construction

As shown in Table 1, there were consistent differences between the scene construction scores for the IC and no-IC groups.

A multivariate analysis of variance (MANOVA) showed a highly significant multivariate effect of IC status on scene construction scores, Pillai’s Trace =.509, *F*(4,55) = 14.279, *p* < .001, $\eta_{\text{p}}^{2}\;=\;.509.$ Group differences were observed for all four individual categories: SR, *F*(1, 58) = 14.32, *p* < 0.001, $\eta_{\text{p}}^{2}\;=\;.198;$ EP, *F*(1, 58) = 21.63, *p* < 0.001, $\eta_{\text{p}}^{2}\;=\;.272;$ SD, *F*(1,58) = 24.04, *p* < 0.001, $\eta_{\text{p}}^{2}\;=\;.293$; TEA, *F*(1, 58) = 29.81, *p* < 0.001, $\eta_{\text{p}}^{2}\;=\;.339.$ Notably, all four components—especially TEA—had large effect sizes as shown by partial *η*^2^ (>.198; Cohen, 1988).

To further test the idea that having an IC in childhood is associated with imagination in adulthood, self-rated imagination scores for each group were compared using a Mann-Whitney *U*-Test (used due to non-normal outcome data). The results showed that the IC group (median = 7.5, range 3–9) had significantly higher self-rated imagination scores than the No-IC group (median = 6.0, range 2–10; *U* = 242.50, *Z* = −2.518, *p* = 0.012).

IC status was unrelated to gender, χ^2^(1) = 0.40, *p* = 0.527, and there were no group differences in age, *t* (58) = −0.996, *p* = 0.324.

### Scene Construction and Autobiographical Memory

To assess whether those who had higher scene construction scores also scored higher on the Memory Characteristics Questionnaire, the Pearson’s correlation between total AMT score and total scene construction score was calculated. A marginally significant correlation was found between the two (*r* = 0.25, *p* = 0.05). However, further analysis of the data reveals that, when the scene construction scores were separated into the four individual components, the score for TEA was the only remaining significant association, carrying the overall relationship with total scene construction score (see Table 2).

Given the relation between autobiographical memory and scene construction performance, it was possible that the former could have confounded the comparison of imagination skills between those who did and did not report an IC. Thus, the comparison of scene construction scores was rerun including autobiographical memory (AMT) scores as a covariate in a MANCOVA. This analysis produced almost identical results. In addition, AMT scores were not related to age, *r* = 0.008, *p* = 0.950, nor were there any group differences in AMT between genders, *t*(58) = 1.382, *p* = 0.172, or between those with and without an IC, *t*(58) = 1.45, *p* = 0.152.

## Discussion

Our findings offer support for the first hypothesis: Compared to those not reporting a childhood IC, participants reporting an IC performed better on the scene construction task and rated their own imagination ability more highly. Only partial support was found for the second hypothesis, with the TEA scene construction component being the only component to be positively associated with AMT scores.

### Childhood Imaginary Companions are Associated With Imagination Skills in Adulthood

Seiffge-Krenke’s (1997) *giftedness hypothesis* proposed that inventing a fictitious character is a sign of a particularly creative and imaginative individual. Previous research into this theory has suffered from the methodological challenges inherent in assessing childhood imagination (Taylor, 1999). This study avoided some such limitations by assessing imaginative abilities through task performance rather than self-report, confirming previous findings (e.g., Gleason et al., 2003; Kidd et al., 2010; Dierker et al., 1995) that retrospective reports of childhood ICs are associated with imagination competence in adulthood.

The view that childhood engagement with an IC predicts imaginative competence in adulthood was also supported by participants’ imagination self-rating scores. Although only a very simple 10-point scale was used, the IC group reliably reported themselves as being more imaginative than their No-IC counterparts. This result lends support to Kidd et al.’s (2010) finding that those reporting a childhood IC rated themselves higher on a creativity scale, and also accords with previous research showing that those in professions concerned with creativity and imagination (such as fiction writers) report a higher percentage of childhood ICs than the general population (Myers, 1979).

### The Link Between Thought/Emotion/Action Statements and Autobiographical Memory

Although a positive correlation between the total scene construction score and the AMT scores was observed, this appeared to depend solely on the TEA component of scene construction. This interesting finding is both contradicted and supported by previous research. Hassabis et al.’s (2007) study found that patients with amnesia who were impaired on the scene construction task produced significantly fewer statements for each category than controls, with the largest between-group difference existing for TEA component. This suggests that this is the area where imagination and memory share the most similarity, although such an explanation is called into question by the findings of Raffard et al. (2010), wherein the TEA component was the only category in which individuals suffering from schizophrenia did not score significantly lower than controls, despite also showing memory impairments.

An alternative explanation for these findings may, therefore, lie in the specific method used to assess memory. Previous explorations of the relation between creating fictitious scenes and recalling past memories have not used self-rating instruments like the Memory Characteristics Questionnaire. This may indicate that our AMT scores did not reflect the richness of an individual’s autobiographical memories so much as the person’s emotional involvement with their chosen memory. Such an explanation is consistent with the known role of emotion in encoding and retrieval (Holland & Kensinger, 2010). This pattern of findings might then be explained in terms of individuals who produce more TEA statements being more naturally inclined to relate to the emotional content of a scene. Consequently, individuals may also be more likely to report higher ratings on the Memory Characteristics Questionnaire where several of the scales are related to personal thoughts and feelings about memories (for example, the extent to which the storyline of the memory narrative is bizarre or realistic, or to which personal feelings about the memory are very intense or not intense).

### Autobiographical Memory and Scene Construction do not Correlate Strongly in a Student Population

The lack of a relationship between AMT and other components of scene construction contradicts previous findings (Raffard et al., 2010; Hassabis et al., 2007). The most likely explanation for this can be found in the composition of the population used in this sample. Previous research in this area has involved participants with some form of memory impairment or deterioration through amnesia, schizophrenia, autism, or old age. This may mean that imagination and memory do, indeed, draw on a similar network of brain regions, but this may only have a bearing upon an individual’s fundamental cognitive ability to produce scenes and recall memories, rather than on the level of detail and richness that they provide. This explanation is supported by Szpunar and McDermott’s (2008) argument that the strong connection between imagining the future and remembering the past is evident in certain populations that lack access to specific details of their past that are necessary to mentally construct future scenes. In a typical young adult population, such access is unlikely to be restricted, suggesting that scene construction competence will be determined by individual differences, nondevelopmental experiential factors, and an individual’s environment, as opposed to their neurological functioning. To give an example, those who are naturally more descriptive in the scene construction task may be avid readers of fiction, helping them to construct a rich scene, but not necessarily making them more likely to remember past events in greater detail.

### Limitations and Future Research

Several study limitations require consideration. First, although age was not a statistically significant confounding variable, this may be because the sample only consisted of students aged 18–23 years. The nature of retrospective reports of ICs means that younger participants may be more likely to remember having an IC. Future research should utilize larger samples of more diverse ages to determine how stable imagination really is across the lifespan.

Second, defining childhood imagination in terms of IC engagement is open to criticism. Taylor (1999) argued that having an IC as a child is by no means the only way that children express their imagination, and some highly imaginative children do not engage in such play. However, as this was a retrospective study, there were few alternative ways of assessing childhood imagination. Additionally, the scene construction procedure relies heavily upon verbal ability, and although the sample used was composed of students educated to a similar level, individual verbal ability may have acted as a confounding variable. This study did not control for language ability or selective language problems, and hence some participants might have been disadvantaged by the choice of methodology.

Limitations also surround how autobiographical memory was measured, mainly because self-rating measures are unavoidably—and by definition—subjective. Some participants may have been more prone to rating their memories more highly on the scales. The relative weakness of this measure may, in part, explain the limited association between memory and scene construction found here. Finally, both the memory and imagination tasks relied on single-item measures, and the present findings should ideally be replicated with multiple measures of each capacity.

Future research in this area would, therefore, benefit from utilizing different indices of imagination and autobiographical memory. A potential way of investigating the validity of scene construction as an assessment of imagination could be by comparing it with performance on a nonverbal measure such as a test of creative imagination (e.g., Karwowski, 2008), which would also remove the potential confound of verbal ability. In addition, a limitation of the scene construction task is that it invites descriptions of physical scenes that do not necessarily have a social component. Future adaptations of the task could include specifically social scenarios, such as imagining saying goodbye to a loved one (see also Hassabis et al., 2014). This would, in turn, illuminate the role of internalized social models in imagination more generally, and specifically in the generation of representations of nonactual social agents that occurs in engagement with ICs (Davis, Meins, & Fernyhough, 2014), and in less typical experiences such as auditory verbal hallucinations (Wilkinson & Bell, in press).

More research is also needed into the relation between imagination and memory in a typical young adult population. First, one might compare scene construction performance and performance on a nonself-rated measure of autobiographical memory. Second, building on previous research with individuals affected by memory impairments, neuroimaging studies with typical adults could be used to determine and differentiate neural areas activated when performing imagination and memory tasks. Studies such as these promise to shed further light on the intriguing developmental relations among creativity, imagination, and memory.

## Figures and Tables

**Table 1:** Mean (Standard Deviation) Scores for Participants With and Without an Imaginary Companion

	IC	No-IC	P
Scene construction (total)	101.8 (21.85)	66.43 (15.71)	<0.001
Spatial reference	9.85 (4.17)	6.38 (2.87)	<0.001
Entity presence	35.7 (9.24)	25.15 (7.77)	<0.001
Sensory description	34.05 (8.64)	24.15 (6.04)	<0.001
Thought/emotion/action	22.2 (11.42)	10.25 (5.61)	<0.001
Autobiographical memory (total)	225.66 (29.23)	214.26 (28.35)	0.152

IC = imaginary companion.

**Table 2:** Pearson’s Correlations Between Scene Construction Scores and Autobiographical Memory

	r	p
Scene construction (total)	0.24	0.050
Spatial reference	0.17	0.189
Entity presence	0.26	0.263
Sensory description	0.07	0.580
Thought/emotion/action	0.37	0.004
